# Perfluorocarbon-Loaded Lipid Nanocapsules to Assess the Dependence of U87-Human Glioblastoma Tumor pO_2_ on *In Vitro* Expansion Conditions

**DOI:** 10.1371/journal.pone.0165479

**Published:** 2016-10-27

**Authors:** Laurent Lemaire, Janske Nel, Florence Franconi, Guillaume Bastiat, Patrick Saulnier

**Affiliations:** 1 INSERM U 1066, ‘Micro et Nanomédecines biomimétiques - MINT‘, Angers, France; 2 Université Angers, UMR-S1066, Angers, France; 3 PRIMEX-IRM, Université d’Angers, Angers, France; Technische Universitat Munchen, GERMANY

## Abstract

Growing tumor cell lines, such as U87-MG glioma cells, under mild hypoxia (3% O_2_) leads to a ca. 40% reduction in growth rate once implanted in the brain of nude mice, as compared to normoxia (21% O_2_) grown cells, wherein the former over-express HIF-1 and VEGF-A. Despite developing differently, the tumors have similar: blood perfusion, oxygen consumption, and vascular surface area parameters, whereas the number of blood vessels is nearly doubled in the tumor arising from normoxia cultured cells. Interestingly, tumor oxygen tension, measured using ^19^F-oximetry, showed that the normoxia grown cells led to tumors characterized by mild hypoxic environment (approximately 4%) conditions, whilst the hypoxia grown cells led to tumors characterized by physioxic environment (approximately 6%) conditions. This reversal in oxygen concentration may be responsible for the apparent paradoxical growth profiles.

## Introduction

Most of the studies dealing with glioblastoma cell lines are performed with cells cultured at atmospheric oxygen concentration (i.e. 21% O_2_), despite the fact that brain tissue oxygenation hosting the aforementioned tumoral cells is always lower, and as soon as the tumor proliferates the pO_2_ decreases further, thus tending towards zero within its necrotic center [1–4].

Over the past years, the issue of the ‘appropriate’ oxygen tension to culture glioma cell lines or glioma stem cells has emerged as an important issue as the phenotype of the cells can be radically modified with oxygenation [5]. Such changes include: the significant alteration of HIF-1 expression in high oxygen concentrations [6,7]; the reduction of CD133 expression [8,9]; alterations in the expression of numerous genes involved in angiogenesis or metabolic reprogramming [10,11] and therefore in cellular metabolic profiles [12,13]; as well as a possible impact on therapeutic intervention, such as photodynamic therapy [14]. However, these modifications can be transitory and may be attenuated or reversed once the cells are implanted in animals for subsequent *in vivo* studies. Indeed, the fate of the cells after stereotactic inoculation in the mouse brain appears heterogeneous. Kathagen et al. [10] described a glioblastoma stem-like tissue cell line from a patient biopsy, cultured under hypoxia (1% O_2_), which grew faster as compared to its culture under atmospheric oxygen tension (21%). The same fate was reported by Bourseau-Guilmain et al. [8] for one grade IV glioblastoma cell line arising from a patient biopsy, but also the reverse situation for another grade IV glioblastoma cell line, i.e. the fastest growth being observed for the cells cultured under atmospheric (21% O_2_) conditions upon implantation in mice. This latest observation was also done with the established U87-MG cell line [13].

Currently, the impact of oxygen tension concentration during cell culture, and the physiology of the subsequent brain tumor, is not extensively documented. With the present study, we are reporting the *in vivo* characterization of a U87-MG tumor model initially expanded under mild (3%) or atmospheric oxygen (21%) conditions, in terms of blood perfusion and oxygenation. Data were obtained using magnetic resonance (MR) imaging, respectively; arterial spin labelling (ASL) [15] and fluorine oximetry (^19^F-oximetry) using dedicated oxygen nanosensors [16].

## Materials and Methods

### Perfluoro-15-crown-5-ether loaded lipid nanocapsules formulation

Perfluoro-15-crown-5-ether (PFCE) loaded lipid nanocapsules (LNCs) (thus PFCE-LNC) were prepared as previously described using a phase-inversion process [16,17]. Briefly, the quantities of the PFCE (oil phase), water and NaCl (aqueous phase), Kolliphor^®^ HS15 and Lipoïd (surfactants) were precisely weighed: m_PFCE_ = 5 g, m_Kolliphor_ = 2.115 g, m_Lipoïd_ = 0.188 g, m_Water_ = 5 g and m_NaCl_ = 0.5 g. A four step process was performed to obtain PFCE-LNCs: to dissolve the Lipoïd within the initial solution, the temperature was increased from standing room temperature to 95°C, where after a 1 minute sonication, using a Microson XL2007 sonication probe set at 10 W (Misonix, Farmingdale, USA) was performed, prior to three heating and cooling cycles from 25°C to 95°C. Upon the final cooling, once the temperature reached 70°C, a rapid cold dilution was performed by adding 3.5 mL of cold (4°C) milli-Q water.

Lipoïd^®^ S75-3 (soybean lecithin– 69% phosphatidylcholine and 10% phosphatidylethanolamine) and Kolliphor^®^ HS15 were supplied by Lipoïd GmbH (Ludwigshafen, Germany) and BASF (Ludwigshafen, Germany), respectively. Perfluoro-15-crown-5-ether (C_10_F_20_O_5_) was provided by Chemos GmbH (Regenstauf, Germany). NaCl was purchased from Prolabo (Fontenay-sous-bois, France). Deionized water was obtained from a Milli-Q plus^®^ system (Millipore, Bilerica, USA).

### *In vitro* conditions for U87-MG cells expansion

All experiments were performed with cells between passages 12 and 15. The U87-MG cell line was provided by the ATCC (LGC Promochem, Molsheim, France) and grown in Dulbecco’s modified Eagle’s medium high glucose (Lonza, Verviers, Belgium) supplemented with 10% fetal calf serum (FCS) (HABS; EFS, Lyon, France) and 1% antibiotic solution (Sigma-Aldrich, Saint Quentin Fallavier, France) at 37°C in a humidified incubator under an atmosphere containing 5% CO_2_, and either 3% or 21% O_2_.

### Orthotopic transplantation of U87-MG cells into mouse brain

Animal care and use were in strict accordance with the regulations of the French Ministry of Agriculture and approved by the Pays de la Loire Ethics in Animal Experimentation Committee under project number 01858.03. The manuscript does not contain patient data. Brain tumors were induced via the stereotaxic inoculation of U87-MG human glioma cells in 7–8 week-old female nude mice (Charles Rivers, France) as previously described [18]. Briefly, under Rompun^®^ (Xylazine, Bayer AG, Leverkusen, Germany) and Clorketam^®^ (Kétamine, Vétoquinol, Lure, France) anesthesia, mice were fixed in a stereotaxic holder and placed on a heating pad to maintain the appropriate physiological temperature. Through a 1mm drilled hole in the skull (anterior −0.5 mm, lateral 2.5 mm, depth −3.5 mm according to the bregma), a 5 μL suspension of 7.5 x 10^4^ U87-MG glioma cells, grown at either 3 or 21% O_2_, were injected over a 10-minute period into the caudate putamen of the right hemisphere. After surgery, mice received a single 15μg/kg subcutaneous injection of Vetergesic^®^ (buprenorphin, Sogeval, France) and were monitored daily for mobility and grooming until their participation in the imaging protocol. Two groups of 18 mice were inoculated with cells grown at either 3 or 21% O_2_. Each group was subdivided into two; 6 mice were dedicated to tumor blood flow measurements and histological analysis and 12 mice for tumor oxygenation measurements.

### *In vivo* measurement of brain tumors volume and perfusion

MR imaging was performed using a 7T scanner (Biospec 70/20 Avance III, Bruker Wissembourg, France) equipped with BGA12S gradient system (675mT/m). Animal body temperature was maintained throughout the experiment by hot water circulation in an animal bed. During the MR protocol, mice were anesthetized with 0.5% of isoflurane and respiration was monitored. Tumor volume was assessed over time using a ^1^H cryoprobe and a rapid acquisition with relaxation enhancement (RARE) sequence (TR = 3,200 ms; effective echo time (T_Eeff_) = 21.3 ms; acceleration factor = 4; FOV = 2×2 cm; matrix 256 × 256; nine contiguous slices of 0.5 mm, Nex = 1). Volumes were calculated from manually drawn region of interest. The tumor volume growth curves were subsequently fitted by the method of least squares with an exponential function and the time constant of the exponential converted into a doubling time value [13]. Tumor perfusion was assessed by a segmented Fast Imaging with Steady-State Precession arterial spin labeling sequence (FISP-ASL). Homogeneous radiofrequency excitation was achieved using a proton volume resonator (diameter 87 mm, homogeneous length 80 mm) and signal reception was performed with an actively decoupled phased array surface coil (4 channels). Blood flow was measured from two T1 maps acquired once with slice-selective inversion and once with global inversion [15]. A series of 40 gradient echoes were acquired after the inversion pulse to acquire T1 maps (flip angle = 8°, echo time = 1.8 ms, field of view = 18 mm x 18 mm, matrix size = 128 x 128, excitation hermite pulse duration = 800 μs, inversion hyperbolic secant pulse duration = 15 ms, imaging slice thickness = 1.5 mm, labeling slice thickness = 3.9 mm, with the first echo started 20 ms after the inversion pulse and the duration between each echoes lasting 60 ms). 32 segments were used to fill k-space. A repetition delay of 13 s was introduced after the acquisition of a set of gradient echoes to allow for full relaxation between two inversion pulses. The total measurement time lasted approximately 14 min. Tumoral blood flow (TBF) was calculated with ParaVision 5.1 software (Bruker, Wissembourg, France). At this stage, tumors did not induce any evident discomfort to the mice as assessed by mobility or grooming. At the end of the imaging protocols, the animals were sacrificed.

### *In vivo* measurement of brain tumors oxygenation

When the tumors were approximately 10 μL in volume, the mice underwent an intra-tumoral stereotactic injection of 8 μL PFCE-LNCs, using a convection-enhanced delivery protocol [19]. The fluorine acquisitions were performed using a 40 mm ^1^H/^19^F birdcage. The geometrical parameters were fixed at FOV = 2 x 2 cm, matrix 64 x 36 zero-filled to 64 x 64, slice thickness = 4 mm, and one average was performed. A Half-Fourier Acquisition Single-Shot Turbo Spin-Echo (HASTE) sequence (RARE factor = 36, TE = 4.6 ms, TR = 6 s) with 16 inversion times ranging between 30 ms to 3500 ms was used to produce T1 maps which were converted into tumoral pO_2_ as previously reported [16].

PO_2_ was assessed on anaesthetized mice (0.5% of isoflurane) at rest, i.e. animals breathing air, as well as after a 20 minute challenge with O_2_. Upon switching back to air, tumor pO_2_ was measured over time, and the profile of the pO_2_ change was then fitted with a mono-exponential function whose time constant mainly reflected tumor oxygen consumption [20,21].). At this stage, tumors did not induce any evident discomfort to the mice as assessed by mobility or grooming. At the end of the imaging protocols, the animals were sacrificed.

### Immunofluorescence

Brain cryosections were air-dried, rehydrated in phosphate buffered saline (PBS) and fixed for 10 min in 4% paraformaldehyde (PFA), pH 7.4, at 4°C. To block non-specific binding, the sections were incubated in PBS containing 4% bovine serum albumin (BSA) and 10% normal goat serum. The sections were incubated overnight at 4°C with isotype controls and primary antibodies against endothelial cells (mouse CD31; BD Biosciences) and proliferative cells (Ki67; Dako). Primary antibodies were detected using biotinylated secondary antibody and the signal amplified using streptavidin—fluorescein (streptavidin-FITC) (Dako, les Ullis, France). Nuclei were counterstained with 4',6-diamidino-2-phenylindole (DAPI) (Sigma-Aldrich, Saint Quentin Fallavier, France). Cryosections from 3 mice of each group (3% or 21% O_2_ initially grown U87-MG) were analyzed under a fluorescence microscope (Axioscope 2 optical, Zeiss, LePecq, Germany). The numbers of CD31 positive vessels and Ki 67 positive cells were counted using the MetaView computerized image-analysis system for three brain cryosections per mouse. Two-four fields/cryosection, at × 200 magnification, were randomly chosen in the tumor. The results are expressed as the mean number of positively stained vessels/mm^2^ for each group ±IC95%.

### RNA isolation, cDNA synthesis and quantitative real-time PCR

Total RNAs were extracted from U87-MG glioblastoma tumors using the *RNeasy* Mini *Kit* (Qiagen). Briefly, 1 μg of RNA was reverse-transcribed into complementary DNA using random primers and *Superscript II* according to the manufacturer’s recommendations (Invitrogen, Life Technologies, Thermo Fisher Scientific). Quantitative real-time PCR was performed using the *sybrgreen* Master mix (Thermo Fisher Scientific) and the LightCycler 480 (Roche Diagnostics) according to the manufacturer’s instructions. Primer pairs used for each transcript were specifically designed using Primer Blast software. The relative abundance of mRNA levels was calculated using the 2^−ΔΔCt^ method for each gene using hypoxanthine-guanine phosphoribosyltransferase 1 (HPRT1) gene Ct values for normalization. Five mRNA levels were evaluated: endothelial growth factor A (VEGFA) involved in angiogenesis, the carbonic anhydrase 9 (CA9) and pyruvate dehydrogenase kinase 1 (PDK1) involved in metabolic reprogramming, of urokinase receptor (UPAR) and matrix metalloproteinase 2 (MMP2) involved in invasion and metastasis.

### Statistical analysis

All quantitative data are represented as mean± standard error of the mean, and the statistical significance was determined using *t*-test. Differences were considered significant if the *p* value was < 0.05.

## Results

Growing U87-MG cells under mild hypoxic conditions, prior to cell implantation within mice brain, impacts subsequent growth *in vivo* [13]. In the present study, this observation was confirmed as the tumor volume doubling time after stereotactic implantation in nude mice brain was calculated at 3.7 ± 0.4 versus 2.0 ± 0.2 days (n = 6 in each group, p < 0.001) when cells were proliferated under 3% or 21% O_2_, respectively.

**Fig 1:**
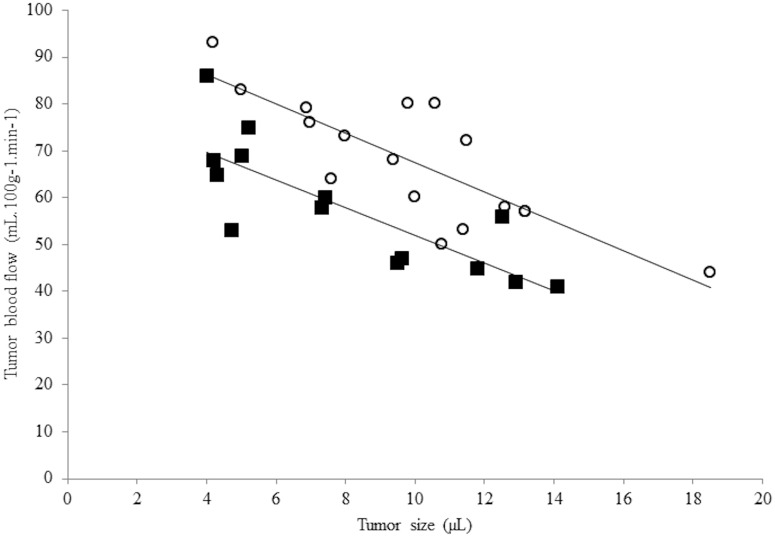
Tumor blood flux as a function of tumor size. This figure illustrates the impact of the tumor size on the tumoral blood flow. Open circles (o) correspond to data acquired in the group arising from the cells initially grown under mild hypoxia (3% O_2_) and close squares (■) to data acquired in the group arising from the cells initially grown under normoxia (21% O_2_).

A fast and non-invasive characterization of the tumor perfusion can be gained using ASL-MR imaging. As shown in Fig 1, it appears that the perfusion is dependent on the size of the tumor in both tumor groups, with a reduction of approximately 3 mL/min/100g of the Tumor Blood Flow (TBF) when the tumor volume increases by 1 μL. As a consequence, tumors were sized matched in the present study. The average tumor size for the hypoxic group (3% O_2_) was 7.1 ± 0.5 μL and 7.5 ± 0.5 for the normoxic group (21% O_2_). Under those circumstances, the measured TBF was not significantly different with values of TBF_3%_ = 69 ± 4 mL/min/100g and TBF_21%_ = 68 ± 5 mL/min/100g for each respective groups (p = 0.79).

**Fig 2:**
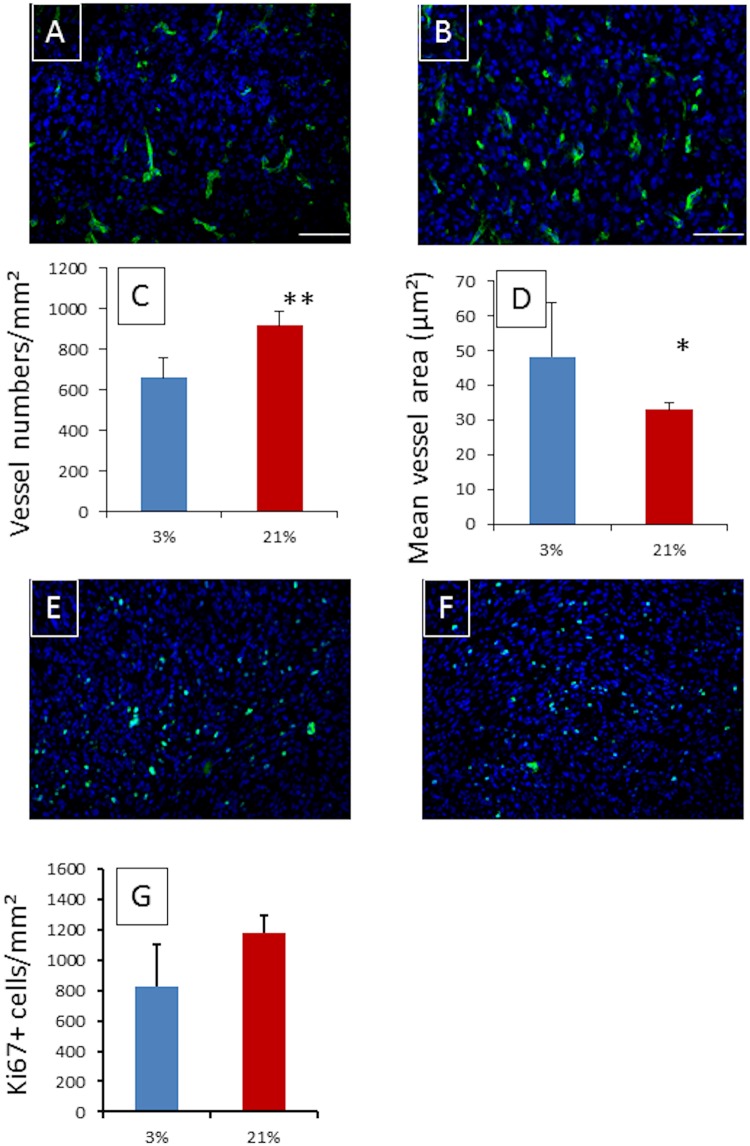
Blood vessel density and cell proliferation assessment with respect to the tumor group explored. Immunofluorescence staining of CD31 and Ki67 in a typical tumor arising from mild hypoxic (3% O_2_) grown U87-MG cells ((A)—CD31 / (E)–Ki67) and from normoxic grown (21% O_2_) tumor ((B)—CD31 / (F)–Ki67) once a size of 8μL is reached. Nucleus staining was performed using DAPI (Blue). Scale bar = 100μm. Quantitative vessel density analysis (C) and average vessel area (D) revealed, on average, 668 ± 50 vessels/mm² with an area of 46 ± 5 μm^2^ in the tumor arising from the cells cultured under mild hypoxia versus 919 ± 35 vessels/mm^2^ with an area of 33 ± 2 μm^2^ in the tumor arising from cells cultured under normoxia [p<0.001 for vessel number (**) and p<0.03 for area (*)]. However, the vascular surface average is not significantly different between both groups and is ~3%. The quantitative analysis of Ki67 positive cells is presented in frame (G), and despite non-significant (p = 0.107) on average, 829 ± 138 positive cells/mm^2^ were counted in the tumor arising from the cells cultures in mild hypoxia condition versus 1178 ± 59 positive cells/mm^2^ in the tumor arising from cells cultured under normoxia.

Nevertheless, despite the absence of difference between both groups in terms of TBF, the histological analysis of the brain tumors showed that the number of vessels was significantly reduced in the 3% group, i.e. n_3%_ = 660 ± 51 vessels/mm² versus n_21%_ = 919 ± 35 vessels/mm^2^ (p < 0.001). Notably, the vessels tended to be larger in the 3% group (p = 0.03), with an average surface area of S_3%_ 48 ± 5 μm^2^ versus S_21%_ 33 ± 2 μm^2^ in the 21% group (Fig 2). Thus the vascular surface area, defined as the number of vessels/mm^2^ multiplied by the average surface of the vessels, is not significantly different within the two types of tumors and represents ca. 3% of the tumoral tissue surface. In terms of the mRNA level of vascular endothelial growth factor A (VEGF-A), carbonic anhydrase 9 (CA9), pyruvate dehydrogenase kinase (PDK1), urokinase receptor (UPAR), matrix metalloproteinase 2 (MMP2), vimentin (VIM) or octamer binding protein 4 (OCT4), an over-expression in the 3% and 21% tumors was measured in comparison to the contralateral brain. However, the comparison between the two groups of tumors only showed a limited 3-fold over-expression in CA9 and a 2-fold over-expression in PKD1, in the 21% O_2_ tumor group as compared to the 3% O_2_.

**Fig 3:**
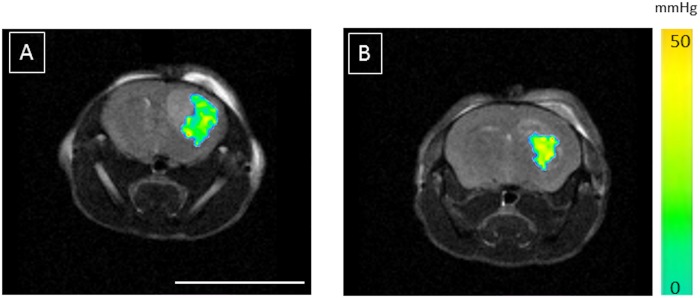
Tumor pO_2_ assessed using perfluorocarbon loaded lipid nanocapsules. The average pO_2_ value of the tumor arising from the cells cultured under mild hypoxia (3% O_2_) (A) is measured at 47 ± 3 mmHg (ca. 6.2% O_2_) whereas a value of 33 ± 3 mmHg is measured (ca. 4.3% O_2_) in the tumor arising from cells cultured under normoxia (21% O_2_) (B) (p <0.01). Those values were measured under isoflurane anesthesia and spontaneous air breathing. Scale bar = 1cm.

In order to assess the tumoral oxygenation, an intratumoral injection of perfluorocarbon-loaded nanocapsules was performed as previously described [16]. The pO_2_ value measured at rest, i.e. isoflurane anesthesia with spontaneous air breathing, within the mice bearing U87-MG brain tumor initially cultured under 3% pO_2_ was equal to pO_2-Air_^3%^ = 47 ± 3 mmHg (n = 12). In the 21% group, the value is significantly reduced (p < 0.01) as pO_2-Air_^21%^ = 33 ± 3 mmHg (n = 12) (Fig 3). After 20 minutes the O_2_ challenge, the measured values for both groups tended towards 100mmHg. Thus fitting the profile of the pO_2_ change over time with a mono-exponential function showed that tumor oxygen consumption, represented by the time constant of the function, is not significantly different (p = 0.4) as values are k_3%_ = 0.35 ± 0.10 min^-1^ and k_21%_ = 0.42 ± 0.13 min^-1^.

## Discussion

The impact of cell phenotype changes, induced by oxygen tension during *in vitro* expansion prior to implantation in living systems, is currently under debate with respect to the pertinence of the models used [5,22], and the potential phenotype reversal/adaptation once the model is hosted in the living environment whose tissular oxygen tension may range from almost 0% up to 15% [3,23]. In the peculiar case of the widely used U87-MG glioma cell line for chemo-therapeutic assessments, cellular therapeutics, and MR methodological purposes [24–26], the impact of the initial culture conditions is often not recognized as a changeable parameter, even though we recently showed that the expansion of this cell line under moderate hypoxia (3% O_2_) or under normoxia (21% O_2_) led to significant modifications in tumor growth [13]. This observation is repeated in the present study. In order to understand the physiological causes of this differential growth, the vascularization of both types of tumor was assessed using non-invasive ASL-MRI and invasive histology. Tumor pO_2_ was also measured using specially designed fluorinated nanosensors [16].

It is now well established that, irrespective of the type of the solid tumor explored, the tumor blood flow (TBF) is dependent on tumor size [27,28]. In this study, this observation was repeated (Fig 1), and in pursuance of comparisons between the two groups, tumors were size matched. Contrary to what Fig 1 portrays, i.e. that TBF tends to be higher in the 3% group as compared to the 21% O_2_ group; the TBF of the tumor sized matched groups at ca. 7–8 μL was not significantly different. Indeed, TBF were measured at TBF_3%_ = 69 ± 4 mL/min/100g and TBF_21%_ = 68 ± 5 mL/min/100g (p = 0.79). Nevertheless, the invasive histological analysis of the tumors showed significant microscopic differences between the two groups. As shown in Fig 2, tumors arising from cells initially grown under mild hypoxia have significantly larger blood vessels, with on average a surface of S_3%_ = 46 ± 5 μm^2^ versus S_21%_ = 33 ± 2 μm^2^ in the 21% group (p = 0.03), but the number of vessels is significantly reduced n_3%_ = 660 ± 51 vessels/mm^2^ versus n_21%_ = 919 ± 35 vessels/mm^2^ (p < 0.001), leading to similar vascular surface area, ca. 3%, possibly explaining why the TBF between the two groups are not dissimilar. However, the vessel density results appear contradictory according to our previous work regarding the *in vitro* evaluation of the hypoxia impact on the cell phenotype [13]. Indeed, *in vitro* mild hypoxia led to the stabilization of HIF-1 and subsequently the over-expression of VEGF-A. As a consequence, we would have expected better angiogenesis in tumors arising from cells initially grown under mild hypoxia. However, a hypothesis involving the oxygen pressure in the host tissue where the cells were implanted, i.e. the rodent striatum, could be proposed. Indeed, a recent report has shown that average oxygen saturation reaches a value of 67% in the rat cortex and 59% in the rat striatum [2,29]. These values can be converted into local oxygen pressures of 50 mmHg (i.e. 6.5% O_2_) and 44 mmHg (5.7% O_2_), respectively, using the Hill equation, wherein n = 2.6 and P_50_ = 38 mmHg [2]. It is therefore possible that an increase in oxygen pressure affecting the cells (rising from 3% oxygen during culturing to ca. 6% in the host tissue) could lead to a switching off of the HIF-1 pathway as previously reported on Hela [30] and Caco2 cells [11]. However, the mirror image of this hypothesis is reflected in the cells cultured under 21% oxygen; even if the oxygen pressure reduction in the host tissue exists, it should not be large enough to trigger the on/off switching of the pathway responsible for the increased angiogenesis. The qPCR analysis performed on the implanted tumors, upon reaching a size of ca. 8 μL, do not provide any support for this hypothesis as no difference in VEGF-A expression is observed between the two groups. However, this hypothesis may be too simplistic; it assumes that the host tissue pO_2_ drives the tumoral cells phenotype from the cell inoculation and as well as throughout the tumor development. As a consequence, we have measured the tumoral pO_2_ using ^19^F-oximetry with specially designed fluorine loaded-nanocapsules [16]. Prior any discussion on the values measured using this method, it has to be noted that the pO2 measurement depend on the infusion zone of the probe within the tumor, and as shown in Fig 3 may not reflect the tumor pO2 of the entire tumor. However, this limitation is common to all methods using exogenous probes, as the distribution within the tumor may be dependent on tissue perfusion as well as on the capillary permeability for example. Nevertheless, we observed that tumors arising from the mild hypoxic cells had a higher pO_2_ (Fig 3), within the sizes used for this study, with values of pO_2-Air_^3%^ = 47 ± 3 mmHg (ca. 6.2% O_2_) versus pO_2-Air_^21%^ = 33 ± 3 mmHg (ca. 4.3% O_2_) (p < 0.01). Moreover, this difference in pO_2_ cannot be attributed to a larger use of oxygen in the 21% tumor group as time constant for this process was defined within an oxygen challenge [21] is no different from the constant calculated for the 3% tumor group (k_21%_ = 0.42 ± 0.13 min^-1^ versus k_3%_ = 0.35 ± 0.10 min^-1^, p > 0.4). Taken all together, the faster growth of the 21% group may result from the tumoral cells creating a local environment, characterized by a lower pO_2_ and transferring the cells into a ‘deeper mild hypoxic’ environment which promotes tumor growth [31], regardless of whether they are implanted in the same physioxic environment as the 3% grown cells. The molecular analysis of the grown tumors cannot unambiguously sustain this observation as it was performed on a single sample from each tumor group. These results, along with the proliferative KI-67 staining experiment, although being not significant (p = 0.107), indicate that the number of proliferative cells is higher in the 21% group, thus adding to the narrative to explain the faster growth of the 21% tumor group.

Following this descriptive study of the macroscopic differences observed relating to hypoxic environments, the next step will be to define the exact biological mechanism(s) and the dynamics which underlays these paradoxical growth profiles as well as a systemic exploration of others glioblastoma cell lines.

## Conclusions

Growing tumor cell lines such as U87-MG glioma cells under mild hypoxia (3% O_2_) or normoxia (21% O_2_) leads to a peculiar phenotype, with over-expression of VEGF-A which should facilitate the growth of hypoxic cells *in vivo*. However, a paradoxical phenomenon is observed as normoxia grown cells gave rise to tumors faster than the mild hypoxia grown cells. Tumor oxygen tension measured using ^19^F-oximetry showed that in the former, mild hypoxic conditions are reached as tumors are growing whereas, for the latter, physioxic conditions are observed with tumor growth and may explain the paradoxical growth profile.

## Abbreviations

^19^F-oximetry: Fluorine oximetry
ASL: Arterial Spin Labeling
BSA: Bovine Serum Albumin
CA9: Carbonic Anhydrase 9
DAPI: 4',6-Diamidino-2-Phenylindole
DMEM: Dulbecco’s Modified Eagle’s Medium
FCS: Fetal Calf Serum
FISP: Fast Imaging with Steady-State Precession
FITC: Fluorescein
HASTE: Half-Fourier Acquisition Single-Shot Turbo Spin-Echo
HIF-1: Hypoxia-inducible Factor 1
HPRT1: Hypoxanthine-guanine Phosphoribosyltransferase 1
Kolliphor: Kolliphor^®^ HS15
Lipoïd: Lipoïd^®^ S75-3
MMP2: Matrix Metalloproteinase 2
OCT4: Octamer Binding Protein 4
PBS: Phosphate Buffered Saline
PFA: Paraformaldehyde
PFCE: Perfluoro-15-crown-5-ether
RARE: Rapid Acquisition with Relaxation Enhancement
TBF: Tumor Blood Flow
VEGF-A: Vascular Endothelial Growth Factor A
